# Social oocyte cryopreservation: a portrayal of Brazilian women

**DOI:** 10.5935/1518-0557.20170024

**Published:** 2017

**Authors:** Elisangela V. Espirito Santo, Felipe Dieamant, Claudia G. Petersen, Ana L. Mauri, Laura D. Vagnini, Adriana Renzi, Camila Zamara, João Batista A. Oliveira, Ricardo L.R. Baruffi, José G. Franco Jr

**Affiliations:** 1 Center for Human Reproduction Prof Franco Jr, Research, Ribeirao Preto, Brazil; 2 Paulista Center for Diagnosis Research and Training, Research, Ribeirao Preto, Brazil

**Keywords:** oocyte cryopreservation, delayed motherhood, fertility preservation, non-medical reasons, social oocyte cryopreservation

## Abstract

**Objective:**

This study aimed to determine what Brazilian childless women of reproductive age think about oocyte cryopreservation to postpone pregnancy and their reasons for performing or not performing this procedure.

**Methods:**

Women of reproductive age were randomly selected from the general population using different e-mail lists and were invited to participate in the study by completing an online web survey regarding social oocyte cryopreservation. The survey was also distributed through social media to women of reproductive age.

**Results:**

Although most of the responders had a partner (86.9%) and had already planned the pregnancy of their first child (69.6%), 85.4% (379) considered the potential of social oocyte freezing to improve their chances of giving birth later in life. Those that had already planned pregnancy were two times more likely to intend to freeze their oocytes (*p*=0.03). The most important barrier for not undergoing oocyte cryopreservation was cost. The women who indicated that they could not currently undergo the procedure now because of cost were two times (*p*=0.03) more likely to intend to cryopreserve their oocytes than women who thought that they would not need to delay pregnancy.

**Conclusion:**

Brazilian women who think that they are not ready to have a family are discovering the option of oocyte cryopreservation. Most participants considered safeguarding their reproductive potential. Making the procedure more accessible could give women the opportunity to make proactive decisions about the future of their fertility.

## INTRODUCTION

Statistics show that the global trends of women choosing to have smaller families and to have families later in life have begun to be felt in Brazil. Brazilian women are having fewer children and, when they become pregnant, they are doing so increasingly later. Already, 30% of Brazilian women have had their first child after age 30 (22.5% in 2000). A public survey identified these changes in the behavior of Brazilian women, relating them to increased years of schooling (Brazilian Health Ministry, 2015). In 2005, almost one-third (30.9%) of the births in Brazil were to women between 20 and 24 years of age. In 2015, there was an increase in childbearing mothers aged 30 to 34 years old (20.3%) and 35 to 39 years old (10.5%). The changes in these age groups were more significant in the south and southeast regions of the country (IBGE, 2015).

Postponing childbearing is linked to a higher rate of involuntary childlessness and smaller families (Schmidt *et al.*, 2012). Women who are aware of the limited female reproductive lifespan have the power to determine and plan their reproductive future. In this sense, oocyte cryopreservation is one available option to prevent age-related fertility decline. Non-medical egg freezing has only been available for about the last 5 years, as new vitrification techniques have made the success rates for actual conception more reliable than the earlier method of slow freezing. The improved outcomes of new technologies of vitrification and intra-cytoplasmic sperm injection have led to the marketing of egg freezing for non-medical reasons, whereby women are offered the possibility of preserving their eggs until such time as they wish to have a child (Allahbadia, 2016).

A survey published in *Fertility and Sterility* observed that the majority of women who froze their eggs reported feeling "empowered", and 53% of them felt more secure about their reproductive future than those who did not cryopreserve their oocytes (Hodes-Wertz *et al.*, 2013). Furthering this debate, since 2014, Apple and Facebook have started offering to freeze eggs for female employees in an effort to attract more women to their staff. This event raised the issue of cryopreservation of fertility, and the media have employed the terms "medical" and "social" to distinguish between the two prevalent reasons for freezing eggs (The Guardian, 2014).

Little is known about the Brazilian context in which women undergo oocyte cryopreservation or their reproductive intentions and perspectives about the procedure. Therefore, this study aimed to determine what Brazilian childless women of reproductive age think about oocyte cryopreservation to postpone pregnancy and their reasons for performing or not performing this procedure.

## MATERIALS AND METHODS

A simple and interactive web survey was designed with a focus on oocyte cryopreservation as a way of delaying childbearing (Table 1). The survey was posted on the Center for Human Reproduction Prof. Franco Junior website (www.crh.com.br/pesquisa), and a message with a link to the website was sent to women of reproductive age, randomly selected using different e-mail lists, to request their participation. The survey was also distributed through social media to women of reproductive age.

**Table 1:** Survey on oocyte cryopreservation

Question	
Age (years)	33.2±6.6
In a stable relationship (Have a partner?)	
Yes	86.9%(386/444)
No	13.1%(58/444)
Would you agree to use donor semen to get pregnant?	
Yes	36.5%(162/444)
No	63.5%(282/444)
Have you already planned when you will have your first child?	
Yes	69.6%(309/444)
No	30.4%(135/444)
Do you know that a woman's fertility decreases after age 35, and that at age 42, the risk of miscarriage is approximately 50%?	
Yes	89.4%(397/444)
No	10.6%(47/444)
Do you know that it is now possible to cryopreserve your eggs to use at a later age through in vitro fertilization?	
Yes	81.5%(362/444)
No	18.5%(82/444)
Would you cryopreserve your eggs to try to get pregnant in the future?	
Yes	85.4%(379/444)
No	14.6%(65/444)
What age do you consider ideal for a woman to cryopreserve her eggs for trying to get pregnant in the future?	
≤35 years	69.8%(310/444)
36- 39 years	24.1%(107/444)
≥ 40 years	6.1%(27/444)
What is the main reason for not cryopreserving your eggs now?	
- Cost	49.3%(219/444)
- It is not necessary	28.6%(127/444)
- Unsatisfactory results	4.9%(22/444)
- Fear of fetal anomalies	2.3%(10/444)
- Ethical and religious reasons	2.3%(10/444)
- Other reasons:	12.6%(56/444)
Educational level	
Secondary education or less	34.2%(152/444)
Bachelor's degree or equivalent	37.2%(165/444)
Master's/Doctoral degree or equivalent	28.6%(127/444)
Income/month *	
≤ $620.00 USD	27.7%(123/444)
-> $1,250.00- ≤ 3,100.00 USD	37.4%(166/444)
-> $1,250.00- ≤ 3,100.00 USD	22.1%(98/444)
> $3,100.00- ≤ 6,200.00 USD	8.3%(37/444)
> $6,200.00 USD:	4.5%(20/444)

* 1 US Dollar (USD) = 2.6 Brazilian Real.

The survey was performed from May 2015 to November 2015, and there was no incentive for completing it. Inclusion criteria were being a childless woman of reproductive age, residing in Brazil and having available Internet access. At the beginning of the survey, participants who answered yes when asked if they have had children were disqualified and automatically excluded from the study. This study was approved by the institutional review board of local ethics committees.

Data management and analysis were conducted using StatsDirect statistical software version 2.7.9 so (Cheshire, UK). A multivariate logistic regression analysis was performed to identify the factors affecting the women's decision-making regarding oocyte cryopreservation.

## RESULTS

Of a total of 583 women who answered the survey, 139 were excluded because they either did not meet at least one of the inclusion criteria or did not answer all the questions. The final sample for this study consisted of 444 women.

Data showed that the study population had reasonable knowledge about the decline of fertility with advanced age (89.4%; 397/444) as well as the best age to cryopreserve their oocytes (≤35 years = 69.8%; 282/444). The study population had negative views about receiving a sperm donation even if that was necessary for them to become mothers (63.5% not accept; 282/444). Although most of the responders had a partner (86.9%; 386/444) and had already planned the pregnancy of their first child (69.6%; 309/444), 85.4% (379/444) considered the potential of social oocyte freezing to improve their chances of giving birth later in life. The most important barrier for undergoing oocyte cryopreservation was the cost (49.3%; 219/444). Table 1 summarizes the answers.

The multivariate logistic regression indicated that those who had already planned pregnancy were two times more likely to intend to freeze their oocytes (OR: 1.95; 95% CI 1.06-3.60; *p*=0.03). An increase in wage level increased the odds of oocyte cryopreservation by 24%. The women who indicated that they could not currently undergo the procedure now because of cost were two times (OR: 2.1; 95% CI 1.07-4.04; *p*=0.03) more likely to intend to cryopreserve their oocytes than women who thought that they would not need to delay pregnancy.

## DISCUSSION

Oocyte cryopreservation has recently become available for patients with concerns about future fertility (Miller & Davis, 2014). In 2012, the American Society for Reproductive Medicine removed the label of "experimental" from this technology, but with a disclaimer that, because there are no data to support the safety, efficacy, ethics, emotional risks and cost-effectiveness of oocyte cryopreservation, there are not yet sufficient data to recommend egg freezing for the sole purpose of circumventing reproductive aging in healthy women (ASRM, 2013). According to Garcia-Velasco *et al.* (2013), oocyte vitrification is a simple, safe, and efficient option to preserve gametes. This can be done for different reasons, from oncological therapies to age-related fertility decline. In any case, we should inform women about their individual chances of oocyte survival, which depends heavily on their age, and the possibilities of having a live birth according to the number of frozen eggs to avoid unrealistic expectations or promises. The efficacy of the technique is limited, particularly with the cryopreservation of oocytes from women aged 35 years or older (von Wolff *et al.*, 2015). Zhang *et al.* (2015) affirmed that it is reasonable to counsel women over 40 years old who have frozen eggs that if they form embryos, each embryo transferred has a very low chance (only 5.3%) of yielding a live birth. This article does not enter into the merits or effectiveness of this technology or promise to enlarge the reproductive options of healthy women whose personal circumstances do not allow them to reproduce in their most fertile years. Our sole purpose was to assess Brazilian women's knowledge, attitudes and intentions towards elective oocyte cryopreservation.

When we think about reproductive health in Brazil, perhaps the most noticeable is the early pregnancy of adolescents, which is a public health problem. Low socioeconomic status and low educational level are factors that contribute to the increase of the incidence of adolescent pregnant women in the country. In Brazil, it is in the social strata with lower purchasing power that the highest fertility rates are found (Santos & Nogueira, 2009). On the other hand, in a large and diverse country, we come across a growing group of women who are prioritizing their studies and career and postponing the pregnancy. The Brazilian Ministry of Health promotes many advertising campaigns warning about teenage pregnancy (Brazilian Ministry of Health, 2015), but with this trend of increase number of "older mother", perhaps the moment indicates the need to implement new public policies, which would consider the characteristics of each region of the country.

In this study population of well-informed women, median age of 33.5, characterized by a higher educational level and a reasonable standard of living (66.8%), most would consider elective egg freezing. The women in this study sample also seemed to know that the best outcomes occur for women who are younger than 35 when their eggs are frozen.

In our survey, however, 52.8% of them stated that the high cost of the procedure is the main reason that prevents them from carrying it out. Even with high schooling, the women in the study have a low economic level. Most of them (38%) live in a monthly income range between USD 620 and USD 1250. Petropanagos *et al.* (2015) observed that it is important to consider the ways in which this technology may work to privilege the reproduction of already privileged women and exclude others who cannot pay for it. Unfortunately, social oocyte cryopreservation is inaccessible to women without substantial financial resources, and it could be misleading to frame this technology as a benefit to all women.

## CONCLUSION

In the modern era, women delay childbearing for a variety of social reasons, and in Brazil, it is no different. Brazilian women who think they are not ready to have a family are discovering the option of oocyte cryopreser-vation. Making the procedure more accessible could give more women the opportunity to make proactive decisions about the future of their fertility. Oocyte cryopreservation is a welcome technology as it provides another option for women who want to have children, but it is expensive and for now, inaccessible for most Brazilians.
